# A grand challenge about multiple metabolic control: health care delivery from tertiary level hospital to primary care health center

**DOI:** 10.1186/1479-5876-10-S2-A22

**Published:** 2012-10-17

**Authors:** Yuqian Bao

**Affiliations:** 1 Department of Endocrinology and Metabolism, Shanghai Jiao Tong University affiliated Sixth People’s Hospital, Shanghai Clinical Center for Diabetes, Shanghai 200233, China

## 

Because of the economic growth and changes in lifestyle, diabetes has become a major public health problem in China. A national study from 2007 to 2008 showed that the age-standardized prevalences of diabetes and prediabetes were 9.7% (10.6% among men and 8.8% among women) and 15.5% (16.1% among men and 14.9% among women), respectively, accounting for 92.4 million adults with diabetes and 148.2 million adults with prediabetes. Data from Shanghai urban communities surveys indicated that the 3-year cumulative incidence rates of diabetes and pre-diabetes were nearly 5% and 11%, respectively. In diabetic patients, the prevalence of diabetic retinopathy was 16.9%. One of five known diabetic (KDM) patients and one of ten newly diagnosed diabetic patients had diabetic retinopathy. Even in the prediabetic group, the prevalence was over 5%. Among diabetes and prediabetes, the prevalence of albuminuria was 25.7% and 12.7%, respectively. The prevalence of peripheral vascular disease in diabetic patients was nearly 15%, much higher than that in prediabetes. Type 2 diabetes and its complications are imposing heavy economic burdens on individuals, families, health systems and countries including China. Since 2003, with the support of Department of Disease Control of Chinese Ministry of Health, Chinese Diabetes Society of the Chinese Medical Association published the Guideline for Diabetes Prevention and Treatment in Chinese. The guideline has been revised twice according to the evidence-based clinical trials. So far, multiple metabolic disorders control of diabetic patients is a global problem. Recently, we developed a Hospital-Community Diabetes Integrated Management to increase “three rates”, which were control rate, screening rate of chronic complication and awareness rate of diabetes knowledge in Shanghai communities. We established collaboration between urban hospitals (Tertiary hospitals) and community health services. The hospital and community health services center have their own responsibilities. Tertiary hospital was in charge of training, establishing management guideline and providing referral platform. The community health services center was in charge of organizing management team and medical record, implementing the management according to the guideline and starting dual referral. Up till now nearly 9,000 high risk individuals completed diabetes screening in 3 communities. And more than 5,000 and medical records were managed by the community health services. The “three rates” improved significantly as consequence.

